# Multi-Target Modulation of Interconnected Pathogenetic Pathways by Natural Bioactive Compounds in Endometriosis

**DOI:** 10.3390/antiox15081003

**Published:** 2026-08-13

**Authors:** Kamila Pokorska-Niewiada, Małgorzata Szczuko, Khasan Kayumov, Katarzyna Janda-Milczarek

**Affiliations:** 1 Department of Toxicology, Dairy Technology and Food Storage, West Pomeranian University of Technology in Szczecin, 71-454 Szczecin, Poland; 2 Department of Bromatology and Diagnostic Nutrition, Pomeranian Medical University in Szczecin, 70-111 Szczecin, Poland; 3 Department of Human and Animals Physiology, National University of Uzbekistan Named After Mirzo Ulugbek, Tashkent 100174, Uzbekistan; qayumovhasan642@gmail.com; 4 Department of Biology, Parasitology and Pharmaceutical Botany, Pomeranian Medical University in Szczecin, 70-111 Szczecin, Poland; katarzyna.janda.milczarek@pum.edu.pl

**Keywords:** endometriosis, natural bioactive compounds, NF-κB, angiogenesis, estrogen signaling, apoptosis

## Abstract

Background: Endometriosis is a chronic estrogen-dependent disease characterized by the presence of endometrial-like tissue outside the uterine cavity. Its pathogenesis involves interactions between inflammatory, angiogenic, hormonal, oxidative stress-related, and cell survival-associated pathways, contributing to lesion development and persistence. Current treatment options are often limited by adverse effects, incomplete symptom control, and high recurrence rates. Methods: This integrative review summarizes the molecular mechanisms involved in endometriosis and the potential role of natural bioactive compounds in their modulation. A literature search was conducted using PubMed, Scopus, and Web of Science, and evidence from experimental, animal, and clinical studies was reviewed. Results: Natural compounds such as curcumin, resveratrol, quercetin, EGCG (epigallocatechin-3 gallate) and genistein have been reported to modulate multiple pathways involved in endometriosis. Their biological activity includes modulation of inflammatory signaling, angiogenesis, estrogen-dependent processes, epithelial–mesenchymal transition, oxidative stress, and apoptosis. While some compounds influence several interconnected pathways, others appear to exert more selective effects. Conclusions: The findings summarized in this review suggest that natural bioactive compounds may influence several interconnected mechanisms involved in endometriosis. Although clinical evidence remains limited, these compounds warrant further investigation as potential adjuncts to current therapeutic approaches.

## 1. Introduction

Endometriosis is a chronic and often debilitating disease affecting approximately 10–15% of women of reproductive age; nevertheless, it remains underdiagnosed and inadequately treated. Beyond its clinical manifestations—such as chronic pelvic pain, dysmenorrhea, dysuria, and infertility—it is increasingly recognized as a multifactorial disorder involving complex interactions between inflammatory, hormonal, angiogenic, oxidative stress-related, and cell survival-associated processes. Despite its high prevalence and substantial impact on quality of life, currently available therapeutic options remain limited in efficacy and are frequently associated with considerable adverse effects or high recurrence rates [1,2].

Current treatment strategies are primarily based on pharmacological and surgical interventions. First-line therapies include nonsteroidal anti-inflammatory drugs (NSAIDs), combined oral contraceptives, gonadotropin-releasing hormone (GnRH) analogs, and progestogens. Although these approaches may provide symptomatic relief, they are often associated with adverse effects such as gastrointestinal disturbances, hepatic and renal dysfunction, weight gain, vasomotor symptoms, and mood disorders. Furthermore, a considerable proportion of patients exhibit an inadequate response to treatment [3,4,5].

Surgical treatment remains an important therapeutic option, particularly in advanced cases or when pharmacological management fails. However, even well-executed surgical interventions do not guarantee long-term disease control. Recurrence rates remain high, reaching approximately 21.5%, while five-year recurrence rates range from 40% to 50%. These limitations underscore the urgent need for alternative or adjunctive therapeutic approaches that are both effective and associated with improved tolerability [6,7,8].

In recent years, increasing attention has been directed toward natural bioactive compounds as potential modulators of molecular pathways involved in endometriosis pathogenesis. Experimental studies suggest that these compounds may influence key processes involved in disease progression, including inflammatory signaling, angiogenesis, oxidative stress, apoptosis, and redox homeostasis [9,10]. Importantly, the therapeutic potential of natural bioactive compounds lies not only in their individual biological effects but also in their capacity to simultaneously modulate multiple molecular targets. Such pleiotropic activity may be particularly relevant in heterogeneous and multifactorial disorders such as endometriosis, in which numerous interconnected pathogenetic pathways contribute to lesion persistence and progression. Although the effects of natural compounds on individual mechanisms involved in endometriosis have been increasingly investigated, these findings are often presented within separate biological contexts. As a consequence, interactions between inflammatory, angiogenic, hormonal, and cell survival-related pathways remain less clearly integrated. A more comprehensive perspective may therefore help to better understand the potential role of natural bioactive compounds in the modulation of disease-related processes. Nevertheless, clinical evidence remains limited, and further studies are required to establish their efficacy, safety, and potential role in future therapeutic strategies [11,12,13].

The aim of this integrative review is to provide a comprehensive overview of the molecular mechanisms involved in endometriosis and to evaluate the therapeutic potential of natural bioactive compounds as modulators of interconnected pathogenetic pathways. Particular emphasis is placed on the relationships between inflammatory, angiogenic, hormonal, oxidative stress-related, and cell survival-associated processes, as well as on the ability of natural compounds to influence multiple targets simultaneously. By bringing together evidence from different biological levels, this review aims to provide a broader perspective on the potential role of natural bioactive compounds in the context of current therapeutic challenges and future treatment strategies for endometriosis [14].

## 2. Materials and Methods

### 2.1. Literature Search

A literature search was conducted using the PubMed, Scopus, and Web of Science databases to identify studies related to natural bioactive compounds and endometriosis. Articles published up to July 2026 were considered.

Search terms combined keywords related to endometriosis (“endometriosis”), natural bioactive compounds (“natural compounds”, “bioactive compounds”, “polyphenols”, “flavonoids”), and the main molecular mechanisms involved in disease pathogenesis, including inflammation, oxidative stress, angiogenesis, estrogen signaling, epithelial–mesenchymal transition, and apoptosis. Boolean operators (AND, OR) were used to refine the search strategy. The reference lists of relevant articles were also screened to identify additional studies.

### 2.2. Eligibility Criteria

Original in vitro, animal, and clinical studies investigating the biological activity of natural bioactive compounds in endometriosis were included. Review articles were used primarily to provide background information and to identify additional original studies. Only articles published in English were considered.

Conference abstracts, editorials, case reports, duplicate publications, and studies unrelated to the scope of this review were excluded.

### 2.3. Study Selection and Data Extraction

Titles and abstracts retrieved from the database search were screened to identify studies relevant to the scope of this review. Potentially eligible articles were then assessed in full. Studies investigating the molecular mechanisms involved in endometriosis or the biological effects of natural bioactive compounds on disease-related pathways were included. Conference abstracts, editorials, case reports, duplicate publications, and studies outside the scope of the review were excluded.

The selected studies were reviewed to extract information relevant to the objectives of this review. This included the investigated compound, its chemical class, experimental model, molecular targets and signaling pathways, biological effects, proposed mechanisms of action, available clinical evidence, and major translational limitations, including reported strategies to improve bioavailability. The extracted information was organized into summary tables and integrated into the narrative synthesis presented in this review.

### 2.4. Data Synthesis and Review Approach

The available evidence was synthesized qualitatively with emphasis on the major molecular mechanisms involved in the pathogenesis of endometriosis and their modulation by natural bioactive compounds. Findings from experimental and clinical studies were analyzed together to identify common molecular targets, biological effects, and potential mechanisms of action.

To facilitate comparison, the reviewed compounds were classified into three groups according to the breadth of their reported activity across the major pathogenetic pathways discussed in this review. This classification was introduced solely to organize the available evidence and should not be interpreted as a ranking of therapeutic efficacy, clinical relevance, or the strength of the available evidence. Owing to the diversity of the included studies, no formal quality assessment or quantitative synthesis was performed.

## 3. Molecular Targets in Endometriosis

The pathogenesis of endometriosis involves multiple interconnected molecular pathways rather than a single dominant mechanism. Processes such as inflammation, oxidative stress, angiogenesis, hormonal signaling, and impaired cell survival collectively contribute to the development and persistence of lesions. Understanding these pathways as components of a broader interacting network is essential for identifying potential therapeutic targets, particularly in the context of the multitarget approaches discussed later [15,16]. Although discussed separately, these mechanisms are functionally interconnected and collectively contribute to lesion development and persistence.

### 3.1. NF-κB Signaling as a Master Inflammatory Hub

The nuclear factor kappa-light-chain enhancer of activated B cells (NF-κB) pathway is one of the key regulators of the inflammatory response in endometriosis, integrating signals originating from oxidative stress and the cytokine milieu. Its activation, induced by ROS (reactive oxygen species) and pro-inflammatory cytokines, leads to increased expression of inflammatory mediators such as IL-6, TNF-α, and COX-2, thereby sustaining chronic inflammation [3,17,18]. Activation of NF-κB not only intensifies the inflammatory response but also affects fundamental cellular processes, including cell proliferation, survival, and invasiveness of endometrial cells. At the same time, inhibition of apoptosis is observed, thereby contributing to the persistence of ectopic lesions [19,20]. Of particular importance is the interaction between NF-κB and estrogen signaling. Estrogen may enhance the activity of this pathway, leading to further amplification of the inflammatory response and establishing a self-perpetuating regulatory loop. From a therapeutic perspective, NF-κB represents an attractive target for bioactive compounds that, through anti-inflammatory and antioxidant effects, may limit the activity of this pathway and its biological consequences [21,22,23,24,25]. Beyond its role in inflammation, NF-κB interacts with pathways involved in angiogenesis, estrogen signaling, and cell survival, highlighting its importance in the complex molecular landscape of endometriosis.

### 3.2. Angiogenesis and the VEGF Axis

Angiogenesis plays a key role in the development and maintenance of endometrial lesions by ensuring an adequate supply of oxygen and nutrients. This process is particularly enhanced under conditions of local hypoxia, a hallmark of the endometriotic microenvironment. In response to hypoxia, HIF-1α is stabilized and regulates the expression of numerous proangiogenic genes, including vascular endothelial growth factor (VEGF). Acting through vascular endothelial growth factor receptor 2 (VEGFR2), VEGF stimulates endothelial cell proliferation and migration, as well as the formation of new blood vessels. Newly formed vessels often exhibit an immature structure and increased permeability, which promotes the persistence of inflammation and further lesion progression [15,16,26]. Angiogenesis is closely linked to other processes involved in endometriosis, particularly inflammation and hypoxia, which together contribute to lesion maintenance and progression.

### 3.3. Estrogen Receptor Signaling

Estrogen signaling is an important component of endometriosis pathogenesis, with particular emphasis on estrogen receptor beta (ERβ) overexpression and disruption of the balance between estrogen receptor alpha (ERα) and estrogen receptor beta (ERβ). A key mechanism involves increased aromatase activity, leading to local estrogen production within endometrial tissues. As a result, elevated estrogen levels are maintained, thereby promoting cell proliferation and lesion persistence. Estrogen signaling is closely linked to inflammation. Estrogen may enhance NF-κB activity, which in turn increases the expression of inflammatory mediators and enzymes involved in estrogen synthesis, creating a positive feedback loop. Natural bioactive compounds may modulate this axis through the regulation of estrogen receptors and steroidogenic enzymes, making it an important therapeutic target [9,27,28,29,30,31,32]. Through its interactions with inflammatory and proliferative pathways, estrogen signaling contributes to several key processes involved in lesion development and persistence.

### 3.4. Epithelial–Mesenchymal Transition (EMT) and Invasion

Epithelial–mesenchymal transition (EMT) is a key mechanism that enables endometrial cells to acquire migratory and invasive properties. This process is initiated, among others, by TGF-β, which leads to the loss of epithelial characteristics and increased cellular motility. The regulation of this process involves transcription factors such as Snail and Slug, which control the transition toward a mesenchymal phenotype. Cellular invasion is further supported by extracellular matrix degradation mediated by metalloproteinases, particularly MMP-2 and MMP-9, enabling tissue penetration and the formation of ectopic lesions [33,34,35,36]. More specialized regulators have also been implicated, including miR-34c-5p, miR-141, ILK, eIF3e, and the TGF-β1/SMAD2 axis, as well as biological factors such as melatonin and LXA4, which may modulate the course of EMT [37,38]. Taken together, these observations indicate that EMT is regulated by several molecular mechanisms acting at different stages of lesion development.

### 3.5. Apoptosis Resistance and Cell Survival Pathways

Endometrial cells in endometriosis show an increased ability to evade apoptosis, enabling their survival and accumulation in ectopic locations. The phosphatidylinositol 3-kinase/protein kinase B (PI3K/Akt) and mitogen-activated protein kinase (MAPK) pathways play a key role in this process by promoting cell proliferation while inhibiting mechanisms leading to cell death. Imbalances between pro- and anti-apoptotic proteins, such as Bax and Bcl-2, together with reduced caspase-3 activity, further promote the persistence of pathological cells [24,39,40,41]. Disruption of apoptotic pathways contributes to prolonged cell survival and resistance to programmed cell death, facilitating the persistence of ectopic endometrial tissue [29,42,43,44]. These alterations are closely linked to other pathogenic processes involved in endometriosis, including inflammation, oxidative stress, and aberrant cellular proliferation.

### 3.6. Integrated Inflammatory–Angiogenic Axis: NF-κB–HIF-1α–VEGF Signaling

The mechanisms described above do not function independently but form a complex, interconnected regulatory network. The NF-κB–HIF-1α–VEGF axis plays a particularly important role by integrating inflammatory signals, hypoxia, and angiogenic processes. Activation of NF-κB increases the expression of inflammatory mediators, which may enhance hypoxia-inducible factor 1-alpha (HIF-1α) stabilization under hypoxic conditions. HIF-1α, in turn, induces VEGF expression, promoting angiogenesis and the formation of new blood vessels [22,45,46,47]. The resulting vascular network facilitates further recruitment of inflammatory cells and maintenance of the inflammatory microenvironment, thereby promoting positive feedback loops. These processes are additionally modulated by estrogen signaling, as well as mechanisms associated with EMT and cell survival [48]. Together, these observations highlight the close relationship between inflammation, hypoxia, angiogenesis, and other processes involved in lesion persistence.

## 4. Results

### 4.1. Natural Bioactive Compounds as Multi-Target Modulators of Pathogenetic Pathways

Natural bioactive compounds are increasingly being considered as potential modulators of endometriosis due to their ability to influence several molecular pathways involved in disease pathogenesis. Many of these compounds affect more than one biological process, including inflammation, oxidative stress, angiogenesis, hormonal signaling, and cell survival. As a result, their biological effects often extend beyond a single molecular target [49,50,51,52]. The multi-target activity of these compounds across key pathogenetic axes is summarized in Table 1 and Figure S1 showing the compound patterns has been added.

### 4.2. Modulation of the NF-κB–Redox–Inflammatory Axis

Bioactive compounds display diverse activity profiles along the NF-κB–redox–inflammatory axis. Some compounds modulate several interconnected pathways, whereas others act primarily on specific components of inflammatory signaling. Polyphenols such as resveratrol, curcumin, and EGCG reduce oxidative stress while simultaneously attenuating NF-κB-mediated inflammatory responses. As a result, they influence several processes involved in lesion development and persistence [129]. Recent studies have further shown that curcumin suppresses STAT3- and JNK-dependent inflammatory signaling while reducing IL-6 and TNF-α expression. In addition, resveratrol attenuates inflammatory responses partly through SIRT1 activation [63,74,130].

Similar effects on oxidative stress and inflammatory signaling have also been reported for myricetin, carnosic acid, and luteolin. These findings further support the role of antioxidant compounds in regulating inflammatory pathways associated with endometriosis. Luteolin has also been shown to modulate NF-κB- and MAPK-dependent signaling pathways involved in the inflammatory response [103,105,127,130].

In contrast, compounds such as baicalein and nobiletin appear to exert more focused effects on selected inflammatory mediators and signaling pathways [112,131]. Overall, these findings indicate that natural bioactive compounds regulate inflammatory processes through diverse mechanisms. Their activity ranges from broad modulation of oxidative stress and NF-κB signaling to more selective effects on individual inflammatory mediators.

### 4.3. Modulation of the HIF-1α–VEGF–Angiogenesis Axis

Natural bioactive compounds exert anti-angiogenic effects by interfering with different stages of the HIF-1α–VEGF signaling cascade. Some compounds, including curcumin, EGCG, and ginsenosides, have been reported to modulate hypoxia-related pathways, including HIF-1α stabilization. Others, such as xanthohumol and nobiletin, primarily affect endothelial cell proliferation, migration, and vascular remodeling. Xanthohumol has also been reported to interfere with VEGF-related signaling and angiogenic responses [11,82,106,128]. This finding supports its potential role in regulating vascular processes associated with endometriosis. Recent evidence further indicates that curcumin suppresses VEGF expression and inhibits the migratory capacity of ectopic endometrial cells. These effects further support its anti-angiogenic activity [64]. Together, these observations highlight the close relationship between angiogenesis, inflammation, and hypoxia in endometriosis [16,64,81,132].

### 4.4. Modulation of Estrogen Signaling

The modulation of estrogen signaling is one of the mechanisms through which bioactive compounds may influence endometriosis-related processes. These effects extend beyond direct receptor interaction and include regulation of aromatase activity and local steroidogenesis. Phytoestrogens such as genistein and daidzein can interact with estrogen receptors. In contrast, compounds such as quercetin and puerarin have been reported to affect pathways involved in estrogen biosynthesis [9,11,79,90]. Puerarin has also been shown to suppress aromatase expression and modulate ERα/ERβ- and ERK-dependent signaling, thereby reducing estrogen-dependent proliferation of ectopic endometrial cells [95].

Recent evidence further indicates that quercetin promotes decidualization and improves endometrial receptivity by modulating estrogen-responsive signaling pathways [76].

Oleuropein has also been associated with the regulation of estrogen-related pathways and apoptotic mechanisms, suggesting effects that extend beyond hormonal signaling alone [104]. However, the biological effects of phytoestrogens may vary depending on factors such as dose, receptor subtype, and the hormonal environment. Therefore, although these compounds show promising activity in experimental models, further clinical studies are needed to better define their therapeutic potential and safety in endometriosis. Given the close relationship between estrogen signaling and inflammatory activity, these findings further support the interconnected nature of the molecular mechanisms involved in endometriosis.

### 4.5. Modulation of EMT and Invasion

Bioactive compounds may influence the invasive behavior of endometrial cells by modulating epithelial–mesenchymal transition (EMT) and related migratory processes. They regulate transcription factors involved in EMT and affect extracellular matrix remodeling and cell adhesion. Compounds such as resveratrol, naringenin, and isoliquiritigenin have been reported to attenuate EMT-related signaling and reduce cellular motility. Resveratrol also suppresses TGF-β- and MMP-9-dependent pathways involved in EMT and tissue invasion [73]. Because EMT is closely linked to inflammatory and proliferative pathways, its modulation may also influence lesion development and persistence [37,133].

### 4.6. Regulation of Cell Survival and Apoptosis

Natural bioactive compounds have been reported to influence pathways involved in both cell survival and programmed cell death. These processes are frequently dysregulated in endometriosis. Compounds such as quercetin, luteolin, and chrysin have been shown to enhance apoptotic activity while reducing pro-survival signaling. Recent evidence further indicates that quercetin promotes apoptosis by modulating the AKT/ERK/p53 signaling axis [76]. These effects are associated with alterations in mitochondrial pathways, including changes in the Bax/Bcl-2 ratio and activation of caspase-dependent mechanisms [134,135]. The effects of these compounds extend beyond apoptosis and include the regulation of cellular proliferation and turnover. In addition, curcumin has recently been shown to suppress the H19/IGF signaling pathway, contributing to reduced proliferation of ectopic endometrial cells [64]. As a result, modulation of cell survival pathways may limit the persistence of ectopic endometrial cells [10].

The clinical relevance of these findings remains dependent on pharmacokinetic factors, including bioavailability, metabolism, and achievable tissue concentrations in vivo [136]. These translational aspects are summarized in Table 2.

## 5. Integrative Model of Multi-Target Modulation in Endometriosis

Endometriosis is increasingly understood not as a result of a single dysregulated pathway, but rather as a condition driven by a network of interacting processes. Inflammation, oxidative stress, angiogenesis, estrogen signaling, epithelial–mesenchymal transition (EMT), and impaired apoptosis are closely linked and tend to reinforce each other, contributing to the persistence of lesions [10,49]. Within this context, natural bioactive compounds do not act on one target only. Many of them influence several key regulatory points at the same time, including NF-κB, HIF-1α, VEGF, and estrogen-related pathways. As a result, these compounds may influence several interconnected pathways rather than a single molecular target.

For instance, changes in NF-κB activity can affect both inflammatory signaling and angiogenesis, while oxidative stress may influence apoptosis as well as hormone-related pathways. As a result, the effects of bioactive compounds often overlap across different levels of regulation [214,220]. The interactions between these pathways are dynamic rather than independent. Because these pathways are interconnected, modulation of one pathway may also influence other processes involved in lesion maintenance. Inflammation may increase oxidative stress and angiogenic signaling, whereas hypoxia promotes HIF-1α activation and VEGF expression. In turn, these changes help maintain the local environment that supports lesion growth and persistence, creating positive feedback loops that further sustain the disease process. The relative contribution of these pathways may also vary depending on the local microenvironment and the biological characteristics of individual lesions [10,49]. Not all compounds discussed in this review appear to act with the same breadth of activity. While some, such as curcumin, resveratrol, quercetin, EGCG, genistein, puerarin, and ginsenosides, have been associated with effects across several pathogenetic pathways, others appear to exert more selective actions. Examples include daidzein, which primarily affects estrogen-dependent mechanisms, xanthohumol, which has been studied mainly in the context of angiogenesis, and luteolin, whose reported activity is largely related to inflammatory signaling. This diversity suggests that natural compounds may differ not only in their molecular targets but also in their potential role within multi-target therapeutic strategies.

From this perspective, their role may be better understood in terms of modulation rather than inhibition. Instead of blocking a single pathway, these compounds may influence several interconnected processes, potentially weakening the feedback mechanisms that sustain lesion persistence. In a heterogeneous and multifactorial disease such as endometriosis, this broader mode of action may be particularly relevant.

## 6. Limitations and Future Research Directions

The available evidence on natural bioactive compounds in endometriosis remains dominated by in vitro and animal studies. Although these studies have provided valuable insights into the molecular mechanisms involved in disease progression, their findings cannot be directly translated into clinical practice. Human studies remain limited, and despite the growing number of preclinical studies, clinical data are still scarce for most compounds.

Interpretation of the current literature is further complicated by substantial differences in experimental design. Studies vary with respect to model systems, treatment duration, administered doses, and evaluated outcomes, making direct comparisons difficult. In addition, the level of evidence is not uniform across compounds. While some, such as curcumin, resveratrol, quercetin, and EGCG, have been investigated extensively, others are supported by only a limited number of studies. Moreover, differences in experimental models, treatment protocols, and outcome measures make it difficult to compare the reported effects across studies. Consequently, apparent differences in efficacy between compounds may partly reflect methodological variability rather than true differences in biological activity.

Another challenge concerns the pharmacokinetic properties of many natural compounds. Poor bioavailability, rapid metabolism, limited absorption, and low tissue concentrations remain important barriers to clinical application. As a result, biological effects observed under experimental conditions may not always be achievable in vivo [10,14,50].

Future studies should focus on strengthening the clinical evidence base and improving the comparability of experimental findings. Another challenge is the lack of standardized outcome measures across studies. Future research would benefit from the use of validated biomarkers related to inflammation, angiogenesis, oxidative stress, and hormonal signaling, including NF-κB, TNF-α, IL-6, VEGF, and estrogen receptor-associated pathways. Such approaches may facilitate comparisons between studies and improve the evaluation of treatment responses. Further work is also needed to evaluate formulation strategies designed to enhance bioavailability.

Future studies should also explore the potential of combination approaches involving natural bioactive compounds and established pharmacological therapies. Such strategies may provide broader modulation of interconnected pathogenetic pathways than single-agent interventions. However, little is known about potential interactions with commonly used treatments, including hormonal therapies and nonsteroidal anti-inflammatory drugs. Further studies are needed to clarify these interactions. Further investigation of interactions between inflammatory, angiogenic, hormonal, and cell survival pathways may help identify the most promising therapeutic targets. The effectiveness of natural bioactive compounds may also differ according to lesion phenotype, disease stage, and individual patient characteristics, highlighting the need for more personalized approaches in future research. Greater methodological consistency across future studies would facilitate comparison of results and strengthen the available evidence.

## 7. Conclusions

Endometriosis is a multifactorial disease in which inflammatory, angiogenic, hormonal, oxidative stress-related, and cell survival-associated processes remain closely interconnected. The interactions between these pathways contribute to lesion development, persistence, and progression.

Natural bioactive compounds have emerged as potential modulators of several mechanisms involved in endometriosis pathogenesis. As discussed in this review, many of these compounds affect more than one molecular pathway, including inflammation, angiogenesis, estrogen signaling, epithelial–mesenchymal transition, oxidative stress, and apoptosis. This broad spectrum of activity may be particularly relevant in a disease characterized by complex interactions between multiple biological processes.

Although most of the available evidence is derived from experimental studies, the findings summarized in this review support further investigation of natural bioactive compounds in endometriosis. Their clinical application will require further validation in well-designed human studies, together with improved strategies to enhance bioavailability and a better understanding of their mechanisms of action.

## Figures and Tables

**Table 1:** Natural bioactive compounds grouped according to the breadth of modulation of pathogenetic pathways involved in endometriosis.

Compound	Chemical Class	Main Molecular Targets/Pathways	Cellular and Functional Effects	Evidence	Pathogenetic Axis	References
**Group I. Broad-spectrum modulators of endometriosis-related pathways**						
Curcumin	polyphenol (curcuminoid)	NF-κB, IKKα/β, STAT3, JNK, COX-2, TNF-α, IL-6, VEGF, H19, IGF signaling	suppresses inflammatory signaling and cytokine production; inhibits angiogenesis, proliferation and migration; promotes apoptosis	in vitro, in vivo, clinical evidence	multiple (NF-κB–redox–inflammatory/HIF-1α–VEGF–angiogenesis/cell survival and apoptosis)	[14,19,53,54,55,56,57,58,59,60,61,62,63,64,65]
Resveratrol	polyphenol (stilbene)	SIRT1, NF-κB, TNF-α, VEGF, TGF-β, MMP-9	suppresses inflammation, angiogenesis, invasion, and proliferation; promotes apoptosis	in vitro, in vivo, clinical evidence	multiple (NF-κB–redox–inflammatory/HIF-1α–VEGF–angiogenesis/EMT and invasion)	[62,66,67,68,69,70,71,72,73,74,75]
Quercetin	flavonoid (flavonol)	ERα/ERβ, PI3K/Akt, ERK1/2 (MAPK), VEGF, p53	suppresses inflammation, proliferation and angiogenesis; promotes apoptosis and decidualization	in vitro, in vivo	multiple (NF-κB–redox–inflammatory/HIF-1α–VEGF–angiogenesis/estrogen signaling)	[75,76,77,78,79,80,81]
EGCG (Epigallocatechin-3 gallate)	catechin (flavan-3-ol)	VEGF, VEGFR2, TGF-β/Smad, MMP-9, ROS	suppresses angiogenesis, oxidative stress, proliferation, migration, and invasion; promotes apoptosis	in vitro, in vivo	multiple (NF-κB–redox–inflammatory/HIF-1α–VEGF–angiogenesis/EMT and invasion)	[82,83,84,85,86,87]
Genistein	isoflavone	ERα/ERβ, NF-κB, COX-2, VEGF	modulates estrogen signaling; reduces inflammation; limits angiogenic activity	in vitro, in vivo	multiple (estrogen signaling/NF-κB–redox–inflammatory/HIF-1α–VEGF–angiogenesis)	[11,88,89,90,91,92]
Puerarin	isoflavone glycoside	ERα/ERβ, aromatase (P450arom), c-Jun/AP-1, ERK (MAPK), cyclin D1, cdc25A, MMP-9, ICAM-1, NF-κB	suppresses estrogen-dependent proliferation, angiogenesis, invasion, and inflammatory signaling; induces G1 cell-cycle arrest and promotes apoptosis	in vitro, in vivo	multiple (NF-κB–redox–inflammatory/HIF-1α–VEGF–angiogenesis/estrogen signaling/EMT and invasion)	[11,22,93,94,95,96]
Ginsenosides	triterpenoid saponins	angiogenesis, ER signaling, apoptosis	modulates vascular and hormonal signaling; promotes apoptosis	in vitro, in vivo	multiple (HIF-1α–VEGF–angiogenesis/estrogen signaling/cell survival and apoptosis)	[97,98,99,100,101]
**Group II. Intermediate-spectrum modulators of endometriosis-related pathways**						
Naringenin	flavanone	TNF-α, MMP-2/9, ROS	reduces matrix degradation; limits invasion; promotes apoptosis	in vitro, in vivo	multiple (EMT and Invasion/cell survival and apoptosis)	[23,102]
Myricetin	flavonoid (flavonol)	NF-κB, PI3K/Akt, ROS	reduces oxidative stress; inhibits inflammatory signaling; induces apoptosis	in vitro, in vivo	multiple (NF-κB–Redox–Inflammatory/cell survival and apoptosis)	[103]
Oleuropein	secoiridoid polyphenol	ERβ, caspase-3	modulates estrogen signaling; activates apoptosis	in vivo	multiple (Estrogen Signaling/cell survival and apoptosis)	[104]
Rosmarinic acid	phenolic acid	ROS, NF-κB, inflammatory mediators	reduces oxidative stress; inhibits inflammatory signaling; limits cell proliferation	in vitro, in vivo	multiple (NF-κB–Redox–Inflammatory/cell survival and apoptosis)	[105]
Nobiletin	polymethoxylated flavone	NF-κB, HIF-1α, VEGF	reduces inflammatory signaling; inhibits angiogenesis; limits proliferation	in vitro, in vivo	multiple (NF-κB–Redox–Inflammatory/HIF-1α–VEGF–Angiogenesis)	[106]
Carnosic acid	diterpene phenol	NF-κB, ROS, VEGF	reduces oxidative stress; inhibits inflammatory signaling; limits angiogenesis	in vitro, in vivo	multiple (NF-κB–Redox–Inflammatory/HIF-1α–VEGF–Angiogenesis)	[105]
Ursolic acid	pentacyclic triterpenoid	NF-κB, Bax/Bcl-2, caspase-3	reduces inflammation, induces apoptosis	in vitro	multiple (NF-κB–Redox–Inflammatory/cell survival and apoptosis)	[107]
Dehydrocostus lactone	sesquiterpene lactone	COX-2, PGE2, caspases	inhibits inflammation; activates apoptosis pathways	in vitro	multiple (NF-κB–Redox–Inflammatory/cell survival and apoptosis)	[108]
Apigenin	flavone	NF-κB, TNF-α, Bax	reduces inflammatory signaling; activates apoptosis	in vitro	NF-κB–Redox–Inflammatory	[109,110,111]
**Group III. More selective modulators of endometriosis-related pathways**						
Baicalein	flavone	NF-κB	suppresses inflammatory signaling; inhibits proliferation	in vitro	NF-κB–Redox–Inflammatory	[112]
Chrysin	flavone	PI3K/Akt, ROS	modulates oxidative stress; induces apoptosis	in vitro	cell survival and apoptosis	[113]
Delphinidin	anthocyanidin	PI3K/Akt, ERK	induces apoptosis; inhibits proliferation signaling	in vitro	cell survival and apoptosis	[114]
Ellagic acid	polyphenol	cell cycle regulators	inhibits cell cycle; reduces migration	in vitro	EMT and invasion	[115]
Flavokawain A	chalcone	mitochondrial pathways, caspases, Bax/Bcl-2	induces apoptosis; disrupts mitochondrial function; inhibits proliferation	in vivo	cell survival and apoptosis	[116]
Scutellarin	flavonoid glycoside	angiogenesis	reduces vascularization; limits fibrosis	in vivo	HIF-1α–VEGF–Angiogenesis	[55]
Isoliquiritigenin	chalcone flavonoid	Snail, Slug, MT-related signaling pathways	inhibits EMT; reduces cellular invasion and migration	in vitro, in vivo	EMT and invasion	[117]
Silibinin	flavonolignan	cytokines, oxidative stress	reduces inflammatory response; modulates oxidative stress	in vitro, in vivo	NF-κB–Redox–Inflammatory	[118]
Wogonin	flavone	ERα, cell cycle regulators	modulates estrogen signaling; inhibits cell cycle progression	in vitro, in vivo	estrogen signaling	[105]
β-Caryophyllene	sesquiterpene	apoptosis pathways	promotes apoptosis; reduces lesion viability	in vivo	cell survival and apoptosis	[11,119]
Nerolidol	sesquiterpene alcohol	ROS, mitochondrial pathways, caspases	induces apoptosis; disrupts mitochondrial function; reduces cell viability	in vitro, in vivo	cell survival and apoptosis	[120,121]
Daidzein	isoflavone	Ki-67	reduces proliferation; modulates estrogen-dependent growth	in vivo	estrogen signaling	[122,123,124,125,126]
Luteolin	flavone	NF-κB, MAPK	reduces cytokine production; inhibits proliferation signaling	in vitro	NF-κB–Redox–Inflammatory	[127]
Xanthohumol	prenylated chalcone	PI3K/Akt, VEGF	inhibits angiogenic signaling; reduces vascular formation	in vivo	HIF-1α–VEGF–Angiogenesis	[11,128]

Table note: Compounds were grouped according to the breadth of pathogenetic pathways discussed in this review. The classification reflects the range of molecular mechanisms reported in the literature and does not imply differences in therapeutic efficacy.

**Table 2:** Translational challenges, strategies to enhance bioavailability, current evidence for clinical translation, and future research priorities for natural bioactive compounds investigated in endometriosis.

Compound	Main Translational Limitations	Approaches to Enhance Bioavailability	Current Stage of Clinical Translation	Future Research Priorities	References
**Group I. Broad-spectrum modulators of endometriosis-related pathways**					
Curcumin	poor aqueous solubility, low oral bioavailability, rapid metabolism	nanoformulations, phospholipid complexes, polymeric micelles, lipid-based delivery systems	limited clinical evidence with heterogeneous findings	standardize formulations, optimize pharmacokinetics, and conduct adequately powered multicenter randomized controlled trials	[57,60,137,138,139,140]
Resveratrol	poor oral bioavailability and rapid metabolism	nanoformulations, cyclodextrin complexes, lipid-based delivery systems	limited clinical evidence with heterogeneous findings	optimize dosing regimens, evaluate long-term safety, and conduct larger randomized controlled trials	[141,142]
Quercetin	limited absorption and low oral bioavailability	nanoformulations, glycosylated derivatives, phospholipid complexes	no clinical studies in endometriosis; evidence is restricted to preclinical models	improve bioavailability, characterize pharmacokinetics, and initiate early-phase clinical studies	[78,138,143,144,145]
EGCG (epigallocatechin-3-gallate)	low stability and limited oral bioavailability	nanoencapsulation, lipid-based delivery systems, pro-EGCG derivatives	clinical evidence in endometriosis remains scarce despite promising preclinical findings	evaluate optimized formulations in well-designed clinical studies and establish standardized dosing protocols	[86,146,147,148,149,150]
Genistein	dose-dependent estrogenic activity that may limit clinical application	targeted delivery systems, structural modification	limited clinical evidence with heterogeneous findings	clarify dose-dependent effects, identify appropriate patient populations, and conduct controlled clinical trials	[151,152,153]
Puerarin	limited oral absorption	nanoformulations, absorption-enhancing delivery systems	no clinical studies in endometriosis; evidence is restricted to preclinical models	characterize pharmacokinetics and initiate early-phase clinical evaluation	[154,155,156]
Ginsenosides	variable bioavailability and extensive metabolism	nanocarriers, lipid-based delivery systems	no clinical studies in endometriosis; evidence is restricted to preclinical models	optimize pharmacokinetics and evaluate safety before clinical translation	[157,158]
**Group II. Intermediate-spectrum modulators of endometriosis-related pathways**					
Naringenin	poor aqueous solubility and low oral bioavailability	nanoformulations, lipid-based delivery systems	no clinical studies in endometriosis; evidence is restricted to preclinical models	characterize pharmacokinetics and initiate early-phase clinical studies	[159,160,161,162]
Myricetin	poor solubility, limited absorption and rapid metabolism	nanocarriers, phospholipid complexes	evidence is restricted to preclinical studies	improve bioavailability and validate efficacy in clinically relevant animal models	[163,164,165,166,167]
Oleuropein	limited bioavailability and rapid metabolism	nanoencapsulation, lipid-based delivery systems	no clinical studies in endometriosis; evidence is restricted to preclinical models	evaluate efficacy, safety, and optimal dosing in clinical studies	[168,169,170]
Rosmarinic acid (RA)	limited stability and oral bioavailability	nanoparticle-based delivery systems and structural modification	evidence is restricted to preclinical studies	optimize pharmacokinetics and advance translational development	[9]
Nobiletin (NOR)	poor aqueous solubility	nanoformulations and absorption-enhancing delivery systems	no clinical studies in endometriosis; evidence is restricted to preclinical models	evaluate long-term safety and initiate early clinical development	[171,172,173]
Carnosic acid	limited pharmacokinetic data	nanoformulations	evidence is restricted to experimental studies	characterize pharmacokinetics and validate efficacy in clinically relevant models	[174,175]
Ursolic acid	poor aqueous solubility and low oral bioavailability	nanocarriers, lipid-based delivery systems	no clinical studies in endometriosis; evidence is restricted to preclinical models	develop optimized formulations and evaluate clinical feasibility	[176,177]
Dehydrocostus lactone (DCL)	limited pharmacokinetic and toxicological data	advanced drug-delivery systems	early preclinical stage	conduct toxicological evaluation, pharmacokinetic studies, and validation in animal models	[178,179]
Apigenin	poor aqueous solubility, low oral bioavailability, and rapid metabolism	nanoformulations, phospholipid complexes, and polymer-based delivery systems	no clinical studies in endometriosis; evidence is restricted to preclinical models	optimize pharmacokinetics and initiate early-phase clinical evaluation	[180,181,182]
**Group III. More selective modulators of endometriosis-related pathways**					
Baicalein	poor aqueous solubility and low oral bioavailability	nanoparticles, liposomes, phospholipid complexes	evidence is restricted to preclinical studies	standardize formulations and advance translational research	[183,184,185,186]
Chrysin	poor solubility, low absorption, extensive metabolism	nanocarriers, cyclodextrin inclusion complexes	no clinical studies in endometriosis; evidence is restricted to preclinical models	improve delivery systems and validate efficacy in vivo	[187,188,189]
Delphinidin	chemical instability and poor bioavailability	nanoencapsulation and protective delivery systems	evidence is restricted to preclinical studies	improve stability and advance translational evaluation	[190,191,192,,193,194]
Ellagic acid (EA)	poor aqueous solubility and limited intestinal absorption	nanoparticles, phospholipid complexes	no clinical studies in endometriosis; evidence is restricted to preclinical models	enhance bioavailability and evaluate clinical potential	[195,196,197]
Flavokawain A (FKA)	limited pharmacokinetic and toxicological data	nanoformulations	early preclinical stage	conduct toxicological evaluation and pharmacokinetic characterization	[198,199]
Scutellarin	limited intestinal absorption and poor oral bioavailability	phospholipid complexes, nanoparticle-based delivery systems	no clinical studies in endometriosis; evidence is restricted to preclinical models	optimize drug delivery and evaluate clinical feasibility	[200]
Isoliquiritigenin	low oral bioavailability and rapid metabolism	nanocarriers and lipid-based delivery systems	evidence is restricted to experimental studies	optimize systemic exposure and advance translational studies	[201,202,203,204]
Silibinin/ Silymarin	poor aqueous solubility and variable absorption	phytosomes, nanoparticles, lipid-based delivery systems	no clinical studies in endometriosis; evidence is restricted to preclinical models	develop standardized formulations and initiate early-phase clinical evaluation	[118,205]
Wogonin	limited bioavailability and rapid metabolism	nanoparticles and sustained-release delivery systems	evidence is restricted to experimental studies	optimize pharmacokinetics and advance translational validation	[206]
β-Caryophyllene	limited pharmacokinetic and clinical data	lipid-based delivery systems, nanoemulsions	evidence is restricted to experimental studies	characterize pharmacokinetics and evaluate clinical feasibility	[207,208]
Nerolidol	limited pharmacokinetic characterization	nanoemulsions, lipid-based delivery systems	early preclinical stage	complete pharmacokinetic profiling and efficacy studies	[120,121,209]
Daidzein	variable bioavailability and phytoestrogen- related effects	nanoformulations and targeted delivery systems	no clinical studies in endometriosis; evidence is restricted to preclinical models	clarify dose-dependent effects and identify appropriate patient populations	[210,211,212,213]
Luteolin	poor aqueous solubility and low oral bioavailability	nanoformulations, liposomes	no clinical studies in endometriosis; evidence is restricted to preclinical models	evaluate optimized formulations in clinical studies	[214,215,216]
Xanthohumol	poor bioavailability and extensive metabolism	nanoencapsulation, lipid-based delivery systems	no clinical studies in endometriosis; evidence is restricted to preclinical models	advance clinical development using optimized delivery strategies	[207,217,218,219]

Table note: Poor aqueous solubility, limited oral bioavailability, and rapid metabolism remain the principal barriers to the clinical translation of most natural bioactive compounds. Nanoformulations, lipid-based delivery systems, phospholipid complexes, and other advanced drug-delivery approaches have shown promise in improving systemic exposure and therapeutic efficacy.

## Data Availability

The raw data supporting the conclusions of this article will be made available by the authors upon request.

## Supplementary Materials

The following supporting information can be downloaded at: https://www.mdpi.com/article/10.3390/antiox15081003/s1, Figure S1: Chemical structures of selected natural bioactive compounds investigated in endometriosis.

## Abbreviations

The following abbreviations are used in this manuscript:

COX	cyclooxygenase
EGCG	epigallocatechin-3 gallate
EMT	epithelial–mesenchymal transition
HIF-1α	hypoxia-inducible factor 1-alpha
NF-κB	nuclear factor kappa-light-chain enhancer of activated B cells
VEGF	vascular endothelial growth factor
VEGFR2	vascular endothelial growth factor receptor 2
ROS	reactive oxygen species
MAPK	mitogen-activated protein kinase
ERα	estrogen receptor alpha
ERβ	estrogen receptor beta
